# A Fiber Bragg Grating Interrogation System with Self-Adaption Threshold Peak Detection Algorithm

**DOI:** 10.3390/s18041140

**Published:** 2018-04-08

**Authors:** Weifang Zhang, Yingwu Li, Bo Jin, Feifei Ren, Hongxun Wang, Wei Dai

**Affiliations:** 1 School of Reliability and Systems Engineering, Beihang University, Haidian Dist., Beijing 100191, China; 08590@buaa.edu.cn (W.Z.); liyingwu@buaa.edu.cn (Y.L.); RenFeiFei@buaa.edu.cn (F.R.); dw@buaa.edu.cn (W.D.); 2 School of Energy and Power Engineering, Beihang University, Haidian Dist., Beijing 100191, China; by1504121@buaa.edu.cn

**Keywords:** fiber bragg grating, interrogation technology, peak detection algorithm

## Abstract

A Fiber Bragg Grating (FBG) interrogation system with a self-adaption threshold peak detection algorithm is proposed and experimentally demonstrated in this study. This system is composed of a field programmable gate array (FPGA) and advanced RISC machine (ARM) platform, tunable Fabry–Perot (F–P) filter and optical switch. To improve system resolution, the F–P filter was employed. As this filter is non-linear, this causes the shifting of central wavelengths with the deviation compensated by the parts of the circuit. Time-division multiplexing (TDM) of FBG sensors is achieved by an optical switch, with the system able to realize the combination of 256 FBG sensors. The wavelength scanning speed of 800 Hz can be achieved by a FPGA+ARM platform. In addition, a peak detection algorithm based on a self-adaption threshold is designed and the peak recognition rate is 100%. Experiments with different temperatures were conducted to demonstrate the effectiveness of the system. Four FBG sensors were examined in the thermal chamber without stress. When the temperature changed from 0 °C to 100 °C, the degree of linearity between central wavelengths and temperature was about 0.999 with the temperature sensitivity being 10 pm/°C. The static interrogation precision was able to reach 0.5 pm. Through the comparison of different peak detection algorithms and interrogation approaches, the system was verified to have an optimum comprehensive performance in terms of precision, capacity and speed.

## 1. Introduction

Due to their inherent advantages, such as high sensitivity, compact size, low cost and immunity to electromagnetic interference, FBG sensors have been applied widely in the field of engineering [1], including railway traffic [2], monitoring of cardiac activity [3], fabricating of acceleration sensors [4,5], liquid level sensors for measuring aviation fuel levels [6], humidity sensors [7], strain sensors [8] and so on. The interrogation techniques for FBG sensors have received increasing attention in recent years. The current research status of FBG interrogation may be summarized as follows:

Roy utilized wavelength-modulated tunable distributed feedback lasers and a fiber-optic Mach–Zehnder (M–Z) interferometer to realize low-cost, high-accuracy FBG interrogation [9]. The near-infrared absorption line of methane was utilized as a reference wavelength, which yielded a precision of 1.9 pm. Chen proposed a real-time interrogation system with wavelength division multiplexing (WDM) and frequency division multiplexing (FDM) for a large-scale fiber laser sensor array [10]. In this study, as many as 275 FBG sensors were interrogated, although the final precision was only 3.6 με. Triana designed an interrogation system for multiplexing FBG networks based on the encoding strategy [11]. They provided a new solution for the application of FBG networks in real measurement systems, for which the capacity was approximately three times that of the traditional WDM approach with the precision of 3 pm. Recently, the interrogation technique based on the electro-optic modulator (EOM) could interrogate more than 500 FBG sensors with considerable accuracy [12,13]. Li applied four broad bandwidth FBG sensors to match multiple longitudinal modes with the narrow band-width of F–P laser, before photodetectors (PDs) were utilized to measure the maximum intensity of output to interrogate FBG [14]. A temperature precision of 0.02 °C was achieved with a dynamic measurement range of 24 °C. Han devised an interrogation technique utilizing the intensity detection for an ultra-weak FBG sensing system [15]. This system can achieve a resolution of 222 nε under the dynamic frequency of 2000 Hz by utilizing 342 ultra-weak FBG sensors. Tsai presented an interrogation system based on a free-spectral-range-matching scheme, which reached wavelength precision of 5 pm [16]. In this method, the spectra of FBG sensors were filtered with a F–P filter and classified with a multichannel bandpass filter. Signals were detected by different PDs, which was used to interrogate FBG sensors. Another interrogation method based on a F–P filter was also proposed in a previous study [17], which achieved a resolution of 2.4 pm. A sample of a water-level sensor utilizing FBG sensors was presented by Hongo [18]. The precision of the water-level sensor designed in this paper could reach ±1 cm for a range of 10 m. Dai utilized time-division multiplexing (TDM) and the optical spectrum analyzer (OSA) to interrogate FBG sensors in structural health monitoring [19]. A semiconductor optical amplifier (SOA) was driven by a pulse generator to select the reflected spectrum from a particular sensor, with the precision decided by the performance of SOA. Both Xiao and Yasukazu applied arrayed waveguide gratings (AWGs) in their studies [20,21]. The former study utilized the thermal scanning changing spectrum of an AWG to match the FBG spectrum and achieved a precision of 1 pm, while the latter applied mechanical stretching on FBG sensors to match the AWG spectrum and achieved a higher precision of 0.5 pm. Todd devised an interrogation technique using a scanning filter, M–Z interferometer and a 3 × 3 coupler [22]. The phase shift was employed to interrogate FBG sensors, while a compensation algorithm was utilized to maintain the interferometer drift error at below 0.5%. Yao provided an example of the utilization of a high-speed distributed feedback (DFB) swept laser in FBG interrogation [23]. In this approach, a DFB laser driven by the injection current ramp was used to illuminate FBG sensors. At the same time, a type of incomplete waveform repair algorithm was added to improve accuracy. Anziy proposed a novel type of compact high-resolution multichannel micro-electro-mechanical systems (MEMS)-based interrogator [24], where the linear detector was replaced with a digital micromirror device (DMD). The measured wavelength resolution could reach typically 0.5 pm for an interrogation system with four channels. Posada-Roman proposed a fast interrogator based on electro-optical dual optical frequency combs [25], which improved the minimum detectable ultrasound amplitude of the system to 50 nε. On-chip silicon photonic integration is a promising means to produce an integrated on-chip fiber Bragg grating (FBG) interrogator. Li and Marin separately proposed the interrogators with silicon photonic integration in their papers [26,27]. This might be an important trend in FBG interrogation. In summary, a considerable number of approaches have performed well in terms of interrogation speed, precision or capacity. However, the overall performance of FBG interrogation system should be improved. It is necessary to actualize an interrogation method with high comprehensive performance.

In some application area such as crack measuring and structural health monitoring, high accuracy is demanded when many FBG sensors are interrogated synchronously. To settle this problem and improve the calculation speed, an interrogator with high accuracy, capacity and speed was designed in this article. This research aimed at realizing more than 100 FBG sensors’ interrogation with a precision higher than 1 pm, which could meet most of the requirements of real FBG sensor applications. This is of great significance to the extension of FBG sensors. In addition, this method has reference value for the research of interrogator with high comprehensive performance. The brief introduction of this approach is as below.

Stefani and Anziy introduced the few-mode 850 nm FBG sensor [8,28]. The characteristics of this FBG, such as polarization stability and linearity, were provided in their articles. For this the conclusion that the performances of FBG degrade due to the presence of the higher-order modes can be drawn. Moreover, the sensitivity of FBG is higher when the central wavelength becomes larger according to [8]. Thus, in this paper, the interrogation system for single-mode silica fibers with a central wavelength around 1551 nm was studied. As the peak detection algorithm can influence the precision directly, Anziy proposed a novel dynamic gate algorithm (DGA) for precise and accurate peak detection [29], which uses a combination of a threshold-determined detection window and center of gravity algorithm with bias compensation. Being different from the DGA, a peak detection algorithm based on two types of self-adaption threshold was proposed to suppress noises caused by a broadband light source and electrical circuit in this paper. The proposed system was based on a FPGA+ARM platform, tunable F–P filter and optical switch. For improving resolution, the F–P filter was applied to overcome the low precision of a broadband light source. As FBG has the characteristic of cross-sensitivities [7], a comb filter was employed to compensate for the error of FBG reflection spectrum, which is caused by temperature drift. The optical switch was employed to realize the TDM technique of the optical spectrum and subsequently, enhance the capacity of FBG sensors. A platform combined with FPGA and ARM (zynq7000) was employed to increase the speed of FBG interrogation by improving sampling frequency and computational performance.

The rest of this paper is organized as follows: the interrogation system and interrogation principle are described in Section 2. The self-adaption threshold peak detection algorithm is presented in Section 3. Section 4 presents the experiments and discussion, which includes a brief introduction of the interrogator, comparison of different peak detection algorithm, temperature experiments and the performance of different interrogators. The major findings of this work are summarized in Section 5.

## 2. System Description

The system is described from two aspects: an overview of the interrogation system and the introduction of the interrogation principle. The interrogation system consisted of a FPGA+ARM platform, tunable F–P filter and optical switch. In the optical part, the F–P filter converts a spectrum of broadband light source into a single wavelength spectrum to illuminate FBG sensors and comb filter. In circuit part, the FPGA+ARM platform creates the ramp scanning voltage, which drives the F–P filter. The resolution of F–P filter wavelength scanning was decided by the ramp scanning voltage. As for the interrogation principle, it is based on the tunable F-P filtering principle which is one of the most important and widely utilized methods due to its advantages of simplicity and high precision. By contrasting a FBG reflection spectrum and comb filter transmission spectrum, the central wavelengths can be interrogated. The application of FPGA+ARM platform can make the system have high precision and interrogate large-scale FBG sensors, whilst improving the processing speed.

### 2.1. The Overview of Interrogation System

**Figure 1:**
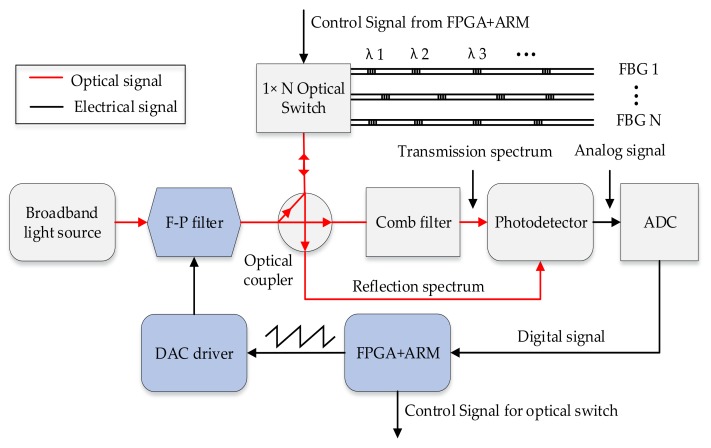
Schematic diagram of the FBG interrogation system.

To interrogate large-scale FBG sensors in the system, we applied an optical switch to accomplish TDM of FBG reflection spectra. The resolution of the system was improved due to the broadband light source being filtered by the 800 Hz scanning F–P filter to produce a scanning single wavelength signal. In addition, the adoption of a FPGA+ARM platform can increase interrogation speed due to FPGA having a high-speed processing performance and ARM having excellent computational performance. The interrogation system was composed of an optical and circuit part, which is schematically depicted in Figure 1.

In the optical part, the F–P filter was adopted due to its advantages of simplicity and high reliability. It provides services for the optical part by converting a spectrum of a broadband light source into a single wavelength spectrum to illuminate FBG sensors and comb filter. However, the production of a scanning single wavelength signal that uses a scanning F–P filter exhibits a non-linear change due to the application of the piezoelectric (PZT) effect, which has the magnetic hysteresis [30]. This causes a deviation of the central wavelengths and thus, the circuit part was employed to compensate the error [31].

**Figure 2:**
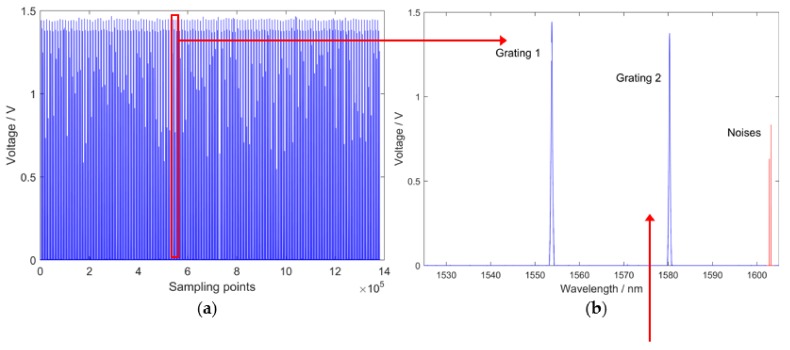

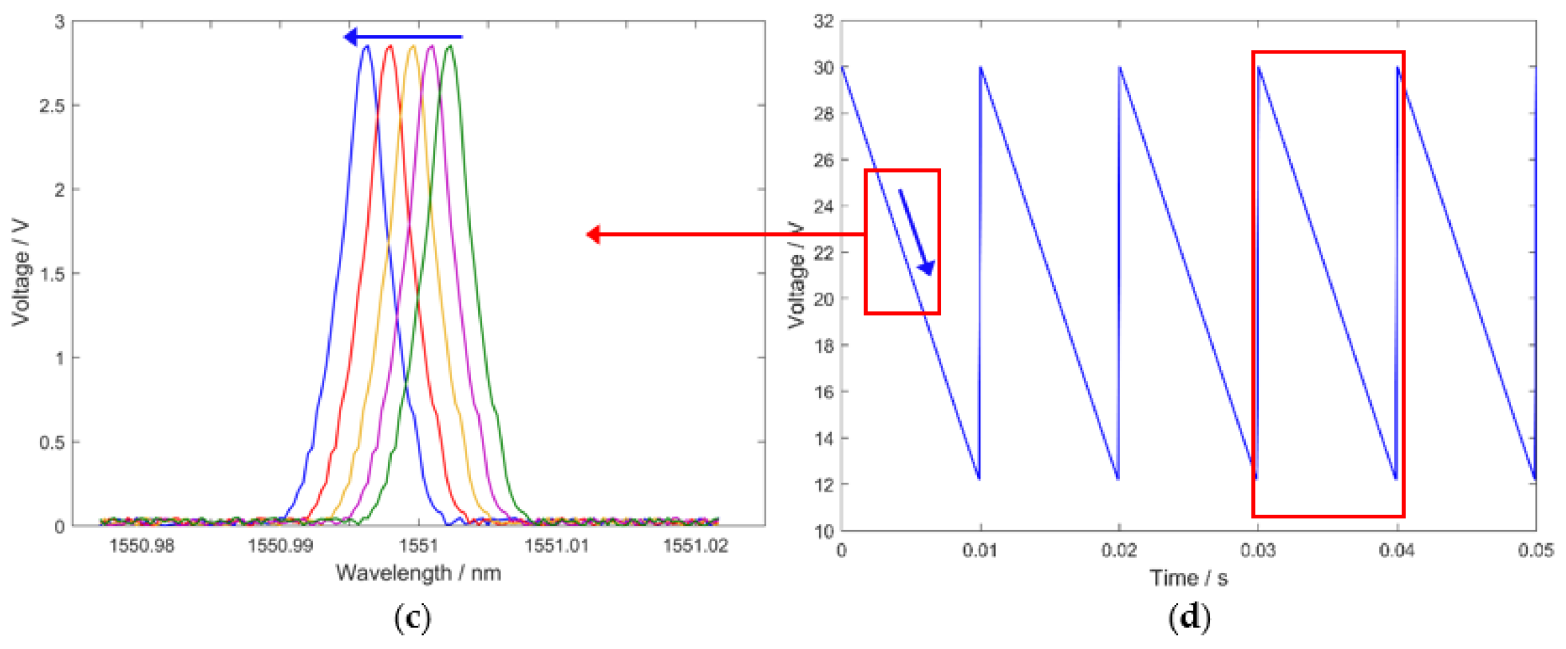
The relationship between key signals: (**a**) the reflection spectrum of the FBG sensor; (**b**) one period waveform of (**a**); (**c**) the output of the F–P filter (different color lines express the corresponding wavelengths when the voltage changes); and (**d**) the 12–30 V ramp scanning voltage signal.

The FPGA+ARM platform controlled the digital-to-analog converter (DAC) to create the ramp scanning voltage, which has a range of 12–30 V. The F–P filter is driven by the ramp scanning voltage signal and its output shift depends on the ramp scanning voltage variation. The period of the ramp scanning voltage signal corresponds to the reflection spectrum. Furthermore, the resolution of F–P filter wavelength scanning was decided by the ramp scanning voltage. The relationships between key signals are shown in Figure 2.

Figure 2b represents one period of Figure 2a, which corresponds to one period of the ramp scanning voltage signal in Figure 2d. The system resolution, which is namely the resolution of the F–P filter output, is proportional to the resolution of ramp scanning voltage (Figure 2c,d). The theoretical resolution of the system can be calculated by Equation (1):

(1) $$\delta_{w}=\frac{W_{max}-W_{min}}{V_{max}-V_{min}}\delta_{v}$$

where $\delta_{w}$ expresses the theoretical resolution of the system; $\delta_{v}$ is the resolution of the ramp scanning voltage; $W_{max}$ and $W_{min}$ are the maximum and minimum wavelengths of the broadband light source; and $V_{max}$ and $V_{min}$ are the maximum and minimum voltages of the ramp scanning signal.

To improve the resolution of the ramp scanning voltage, an 18-bit DAC was adopted. The high-speed characteristic of FPGA can allow DAC to create a ramp scanning voltage with smaller voltage steps in the same amount of time. Due to the range of voltage being 12–30 V, the resolution of ramp scanning voltage can reach $6.9\times 10^{-5}$ V, while the corresponding interrogation range is 1527.0–1602.6 nm. Theoretically, the systematic wavelength resolution can reach 0.3 pm according to Equation (1). Nevertheless, the situation is complicated in practical applications. As depicted in Figure 2b, a two-grating FBG sensor was interrogated and there was impulse noise in the spectrum. To ensure high accuracy, speed and capacity of the system, an algorithm was proposed and a FPGA+ARM platform was applied to accomplish peak detection and interrogation.

### 2.2. The Interrogation Principle

**Figure 3:**
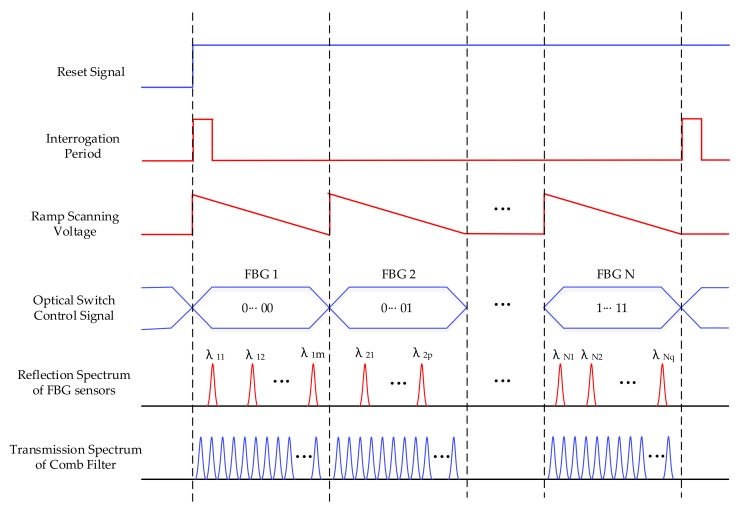
Typical sequence diagram of the system.

A typical sequence diagram of the system is illustrated in Figure 3. With the rising edge of the reset signal and interrogation period signal, the ramp scanning voltage was created by DAC, which has a period that changes with the wavelength scanning period. To improve the capacity of the system, the optical switch was utilized to accomplish TDM of FBG sensors. The optical switch control signal allows access to the interrogation system by specific FBG sensors according to the scanning period. In addition, the FBG reflection spectrum and comb filter transmission spectrum were sampled by an analog-to-digital converter (ADC). The employment of the comb filter can compensate for the error of the FBG reflection spectrum, which is caused by temperature drift. As the transmission spectrum drifts the same way as the reflection spectrum when temperature changes, the comb filter has a similar range of wavelength. Thus, by the contrasting comb filter transmission spectrum and FBG reflection spectrum, the deviation can be eliminated.

**Figure 4:**
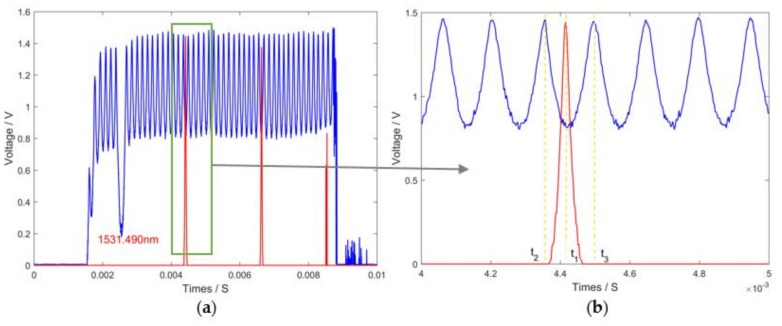
Diagram of the interrogation principle: (**a**) the original reflection spectrum of FBG sensor (in red) and transmission spectrum of comb filter (in blue); and (**b**) the detail diagram of the first grating in (**a**).

Every single peak in the transmission spectrum of the comb filter has a definite wavelength. Using the reflection spectrum of FBG sensors and the transmission spectrum of the comb filter, central wavelengths can be calculated. The transmission spectrum of the comb filter was applied as a reference signal in the system. When the peaks of the reflection spectrum and transmission spectrum are in an identical position, the central wavelengths of the reflection spectrum are equal to wavelengths of the transmission spectrum. Otherwise, because of the linear scanning of single wavelength signal achieved by F-P filter, a linear fitting equation could be utilized to obtain central wavelengths, which is described in Equation (2):

(2) $$\lambda_{FBG}=\lambda_{k}+(\lambda_{k+1}-\lambda_{k})\frac{t_{2}-t_{1}}{t_{2}-t_{3}},$$

where $\lambda_{FBG}$ is the central wavelength of the FBG sensor; $t_{1}$ expresses the time when $\lambda_{FBG}$ appears; $\lambda_{k}$ and $\lambda_{k+1}$ represent the former and the latter peaks of $\lambda_{FBG}$ in the transmission spectrum; $t_{2}$ and $t_{3}$ are the times corresponding to $\lambda_{k}$ and $\lambda_{k+1}$; $\lambda_{k}$ and $\lambda_{k+1}$ are both affirmatory values; and $\lambda_{FBG}$ is matched between $\lambda_{k}$ and $\lambda_{k+1}$. Figure 4 presents the interrogation principle.

FPGA is a type of application-specific integrated circuit (ASIC) and has a distinctive parallel processing system. It is faster than other types of embedded processors in high speed sampling and is suitable for FBG interrogation. Based on the above interrogation principle, the calculation quantity is proportional to the number of central wavelengths. To complete these calculations, FPGA consumes a huge amount of logical resources and time, which reduces the high-speed performance and indirectly decreases the resolution and speed of the system. To address this problem, we employed ARM in an interrogation system to deal with the large number of calculations. ARM uses a reduced instruction set computer (RISC) structure, with a considerable number of registers utilized to support the execution of instruction. This structure provides ARM with a great advantage in terms of data calculation and logical control. ARM has more powerful computational capabilities than FPGA [32,33]. For achieving better performance than the ARM or FPGA platforms, the FPGA+ARM integrated platform (zynq 7000) of Xilinx Inc. (San Jose, CA, USA) was employed in this system. ARM was employed to deal with the large amount of logical control and calculation, while FPGA was applied to realize high speed sampling.

## 3. Peak Detection Algorithm

A peak detection algorithm is proposed in this paper to improve the accuracy of interrogation. It consists of two parts, the improvement of the spectrum waveform and the peak detection algorithm based on self-adaption threshold. The first part utilizes a first order lag filter to inhibit the noises in original spectrum, which is the basics of second part. The second part is utilized to find the position of peaks based on a self-adapting width and height threshold. This peak detection algorithm has advantages of high precision as well as less calculation. Accordingly, this approach is easy to achieve in FPGA+ARM platform by programming.

### 3.1. The Improvement of Spectrum Waveform

**Figure 5:**
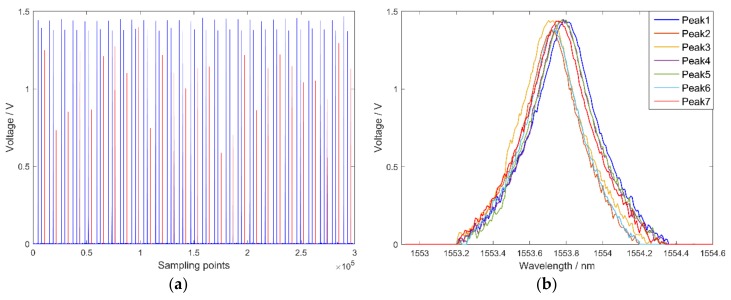
The waveforms of the FBG sensor: (**a**) original reflection spectrum of the FBG sensor; and (**b**) comparison of the FBG sensor’s seven actual peaks.

In reference [23], the interrogation system was based on a high-speed distributed feedback swept laser with incomplete waveforms of the reflection spectrum. Thus, pertinent repair algorithms were proposed for completing waveforms, which improved the interrogation accuracy. In this paper, due to the high-resolution wavelength scanning control for the broadband light source being realized by the F–P filter, the waveforms of the optical spectrum are all complete. Together with amplification and filtering of the analog spectrum signal in the circuit part, the peaks in the original sampling signal show a high degree of similarity. However, high frequency noise (pulse interference and white noise) was detected. The original reflection spectrum of FBG is shown in Figure 5a, in which the blue lines are actual peaks and red lines are pulse interference. Although actual peaks are regular, the existence of pulse interference brings disorder into the reflection spectrum. For comparison, seven actual peaks are selected. As shown in Figure 5b, the waveforms are all complete although they also contain white noise.

To filter out high frequency noise, a first order lag filter was chosen because it possesses the advantages of easy actualization and less computation. At the same time, its hysteresis effect shortcoming can be compensated by the interrogation principle introduced according to Equation (2). The first order lag filter can be accomplished as follows:

(3) $$Y\left(n\right)=\left(1-\alpha \right)X\left(n\right)+\alpha Y\left(n-1\right),\;n\in N^{*}$$

where n is the sequence number of the current sampling point; n−1 expresses the sequence number of the previous sampling point; Y(n) represents the current output of the filter; α is the coefficient of the first order lag filter; X(n) denotes the spectrum’s value of the current sampling point; and Y(n−1) expresses the filtering result of the previous sampling point.

**Figure 6:**
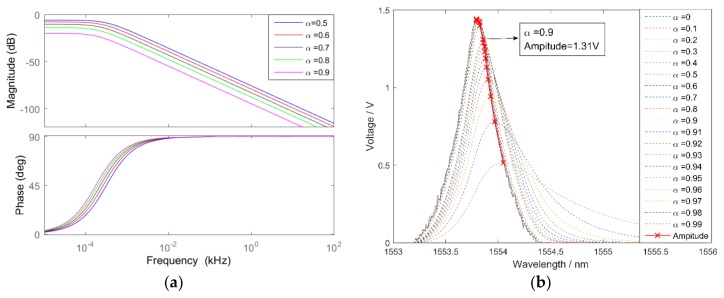
The effect of the first order lag filter: (**a**) the bode plot of the first order lag filter; and (**b**) the peak amplitude after filtering with different α.

**Figure 7:**
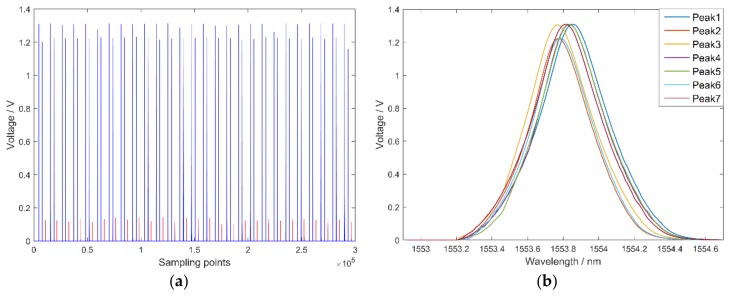
The waveforms of the FBG sensor after filtering: (**a**) reflection spectra of the FBG sensor after filtering; and (**b**) waveforms of seven peaks after filtering.

To obtain the influence of frequency and coefficient α, the Bode plot of the first order lag filter was drawn in Figure 6a. With an increase in frequency and α, the magnitude reduces sharply and phase lags distinctly, which demonstrates that the first order lag filter can inhibit high-frequency noise, although this causes hysteresis of peaks in the spectrum. As α increases, the performance of noise suppression improves. Peak amplitude decreases with an increase in α, with peak amplitudes after filtering with different α depicted in Figure 6b. In this paper, α=0.9 was utilized and the peak amplitude was 90% of the original signal. Reflection spectra and waveforms of peaks after filtering are shown in Figure 7. The pulse interference is significantly reduced and reflection spectra are more regular, while the white noise of peaks is filtered out.

### 3.2. Peak Detection Algorithm Based on the Self-Adaption Threshold

We utilized the Gaussian fit to prove the similarity of peaks as they have consistency in terms of shape. The Sum of Squares due to Error (SSE) and Root Mean Squared Error (RMSE) represent the effect of Gaussian fit. When SSE and RMSE are closer to 0 and the fitting effect is better. The results shown in Table 1 demonstrated that peaks of the reflection spectrum can be fitted by the Gauss model. Accordingly, the similarity of peaks is proved.

**Figure 8:**
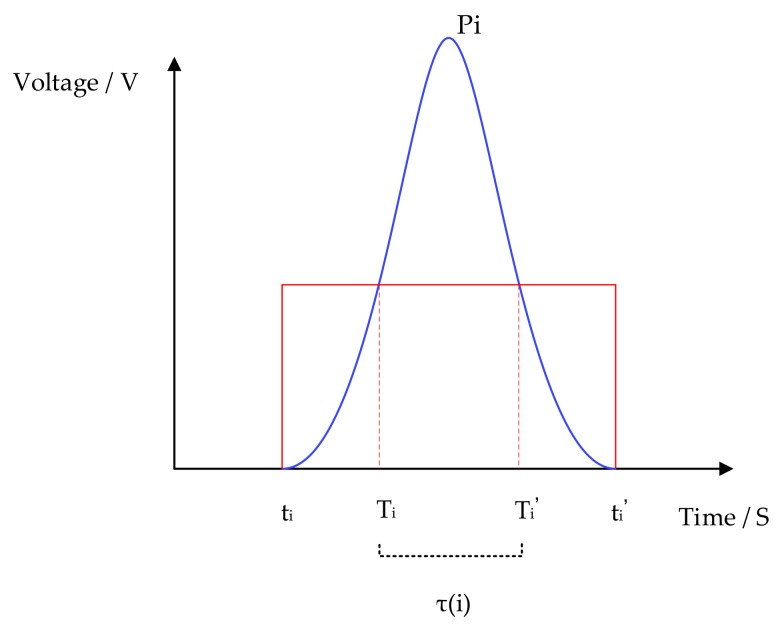
The parameters of $p_{i}$.

In this paper, we proposed a peak detection algorithm based on a self-adaption threshold. With the similarity of the reflection spectrum, this algorithm employed features of known peaks to estimate the subsequent peak. To detect peaks in the reflection spectrum, the peak detection algorithm has two types of thresholds. The height threshold ε(m) is applied to judge the existence of peaks, while the width threshold φ(m) is applied to pick out real peaks, with m being the number of peaks. We hypothesized that $p_{1},p_{2},\cdots ,p_{m},\cdots ,p_{n}$ ($n\in N^{*}$) are peaks in the reflection spectrum of FBG. To obtain the thresholds of $p_{m}$, the parameters of previous peaks should be utilized in the calculation, with the start time of sampling defined as the zero of the time. Figure 8 shows the parameters of $p_{i}$.

The peak can be regarded as the real peak of the reflection spectrum only when the output value of the first order lag filter is larger than ε(m) and the width of the peak is wider than φ(m). ε(m) and φ(m) can be expressed by Equations (4) and (5):

(4) $$\varepsilon \left(m\right)=\rho \left[\frac{\int_{t_{m-1}}^{t_{m-1}^{\prime }}p_{m-1}}{t_{m-1}^{\prime }-t_{m-1}}+\varepsilon \left(m-1\right)\right]\;t_{m-1}^{\prime }>t_{m-1}\geq 0,$$

(5) $$\{\begin{array}{c} \varphi \left(m\right)=\gamma \cdot \sqrt{\frac{1}{m-1}\overset{m-1}{\underset{i=1}{\sum }}{\left[\tau \left(i\right)\right]}^{2}}\;m\leq N\\ \varphi \left(m\right)=\gamma \cdot \sqrt{\frac{1}{N}\overset{m-1}{\underset{i=m-N}{\sum }}{\left[\tau \left(i\right)\right]}^{2}}\;m>N\\ \tau \left(i\right)=T_{i}-T_{i}^{\prime } \end{array},$$

where $t_{m-1}$ and $t_{m-1}^{\prime }$ are the initial and terminal time of the peak $p_{m-1}$; ε(m−1) is the height threshold of $p_{m-1}$; ρ and γ are safety margin coefficients, with ρ,γ∈(0,1); τ(i) is the width of $p_{i}$; *N* is the number of previous peaks calculated; and $T_{i}$ and $T_{i}^{\prime }$ are the initial and terminal time of the peak width.

**Figure 9:**
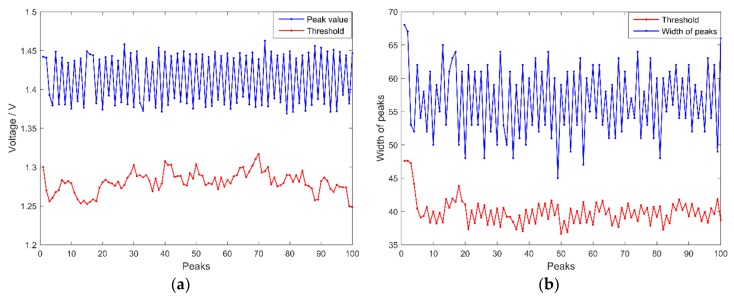
Self-adaption thresholds changed with the variation of 100 peaks: (**a**) ε(m) changed with variation of height; and (**b**) φ(m) changed with variation of width.

The peak detection algorithm based on a self-adaption threshold utilized characteristics of existing peaks to predict the following peaks. These thresholds can adapt themselves to a change in the data. In this paper, parameters were set to N=3, ρ=0.67 and γ=0.74. The peak recognition rate $p_{r}$ is defined by Equation (6):

(6) $$p_{r}=\frac{n_{r}}{n_{a}},$$

where $p_{r}$ is the peak recognition rate; $n_{r}$ is the number of recognized peaks; and $n_{a}$ is the number of actual peaks. The $1.4\times 10^{6}$ sampling data (Figure 2a) were adopted as the input of the peak detection algorithm. As a result, all 250 peaks were detected correctly and thus, the peak recognition rate was 100%. The self-adaption thresholds changing with the width and height obtained from 100 peaks are shown in Figure 9.

## 4. Experiments and Discussion

**Figure 10:**
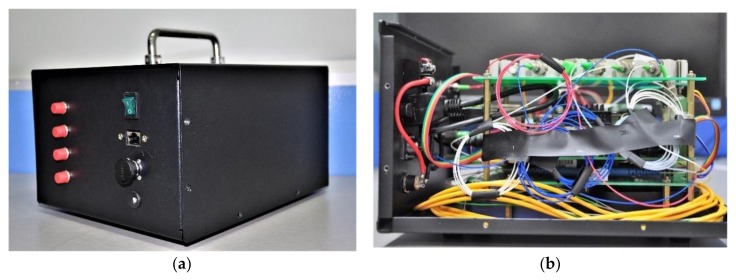
The FBG interrogation system: (**a**) external structure of the FBG interrogation system; and (**b**) internal structure of the FBG interrogation system.

To verify the approach proposed in this article, the system using the above approaches was established with a FPGA+ARM integrated development platform (zynq7000), which is shown in Figure 10. The frequency of FPGA was 100 MHz, the ARM was a dual-core processor and the frequency of each core was 866 MHz. FBG sensors with a Poisson ratio of 0.42, radius of 63.5 µm and central wavelength at around 1551 nm were employed. The DAC driver was a DAC9881, which provided 18-bit resolution and an input data clock frequency up to 50 MHz. The ADC driver, which was an ADS5263 with 18-bit resolution, provided sampling frequencies up to 100 MHz. Four 1 × 4 optical switches were employed with a switching time of 1 ms. The broadband source we used in the system was OS-ASE-M2-CL-F-20-0-S-FA made by Conquer Optics Inc. (Beijing, China), which has a central wavelength range of 1527.0–1602.6 nm and optical power of 20 mW. The 2 × 2 optical splitter we used in the system was made by Conquer Optics Inc., which has a splitting ratio of 2:8 and additional loss of below 0.1 dB. The 80% light intensity was used to illuminate the FBG sensors, while the other 20% was used in the comb filter. The comb filter we used in the system was OS-50G-C made by Conquer Optics Inc., which has a maximal optical power of 500 mW. The F–P filter was TF2D4Z, the free spectral range was 99.9 nm, scanning speed was 800 Hz, insertion loss was 1.91 dB, bandwidth was 13.8 GHz (110 pm) and polarization-dependent loss was less than 0.2 dB.

### 4.1. The Performance of the Peak Detection Algorithm

**Figure 11:**
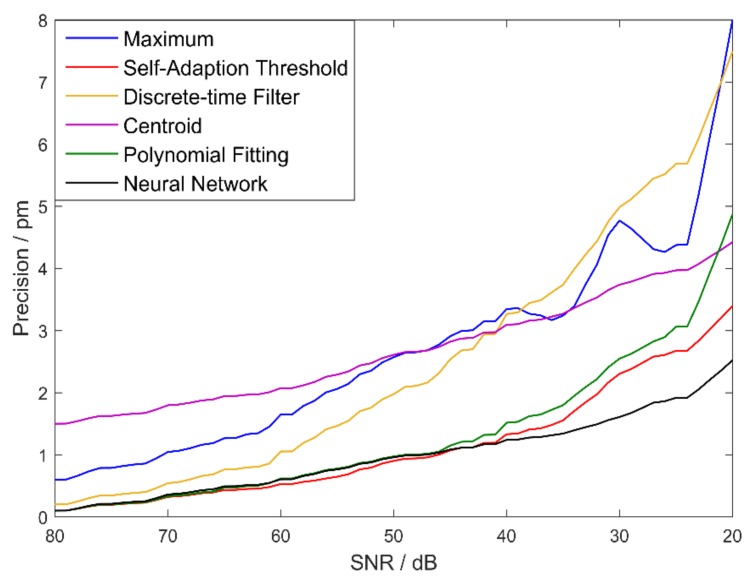
Precision of six peak detection algorithms changing with the variation of SNR.

A considerable amount of research has been conducted on peak detection using typical algorithms, including the maximum algorithm, discrete-time filter algorithm, centroid algorithm, polynomial fitting algorithm and neural network algorithm [34]. The peak detection is very important for FBG interrogation because its properties influence system performance directly. We applied precision and calculation performance to quantitatively analyze the typical algorithms, with six algorithms examined in the FPGA+ARM platform. To obtain the precision of six algorithms under different signal-to-noise (SNR) ratios, white noise was added to the sampling signal, with the results shown in Figure 11. Additionally, execution time and the needed logic unit of six algorithms in the FPGA+ARM platform were used to express the calculation performance, which is shown in Table 2. All values were normalized using the maximum algorithm. As a reference, for the maximum algorithm, the execution time and the needed logic unit were about 0.27 μs and 780, respectively.

As depicted, the precision of all peak detection algorithms decreases with a reduction in SNR. The polynomial fitting algorithm, neural network algorithm and self-adaption algorithm perform better than the maximum algorithm, discrete-time filter algorithm and centroid algorithm. When SNR is greater than 40 dB, the effect of the polynomial fitting algorithm, neural network algorithm and self-adaption algorithm are roughly equivalent. For the implementation of algorithms in embedded hardware, the calculation performance of algorithms is also a critical factor. By contrasting execution time and the needed logic unit, the self-adaption algorithm has preponderance of calculation performance compared to the neural network algorithm and polynomial fitting algorithm. Accordingly, through synthetical consideration of precision and calculation performance, the self-adaption algorithm proposed in this paper performs better than other algorithms.

### 4.2. The Temperature Experiment

**Figure 12:**
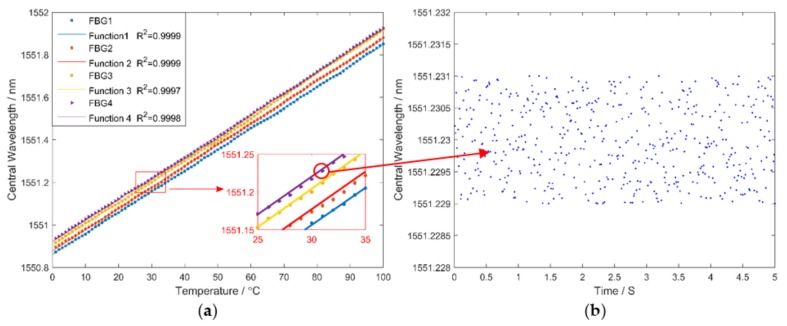
The relationship between central wavelengths and temperature: (**a**) central wavelengths of FBG sensors under different temperature; and (**b**) the measurement result when temperature is constant.

A performance study related to the system by experiments at different temperatures was introduced. Four FBG sensors were placed in a thermal chamber without stress, before the temperature was varied from 0 °C to 100 °C in steps of 1 °C. The measurement time was 2.5 min for each temperature. The central wavelengths of four FBG sensors were 1551.23, 1551.25, 1551.28 and 1551.30 nm. The relationship between central wavelengths and temperature is shown in Figure 12, which was obtained when the temperatures stabilized. As shown in Figure 12a, the central wavelengths of FBG sensors linearly increase with temperature with a temperature sensitivity of 10 pm/°C. In addition, the least square fit was applied to test linearity between temperature and central wavelengths. The results were satisfactory as proved by the goodness of fit, with the degree of linearity being 0.9999, 0.9999, 0.9997 and 0.9998, respectively. When temperature was constant, the mean error of measured wavelengths was used to calculate the precision of the system, which is shown in Equation (7):

(7) $$A=\frac{1}{n}\overset{n}{\underset{i=1}{\sum }}\left|\lambda_{i}-\bar{\lambda }\right|,\;i\in N^{*}$$

where A is the system precision; n is the number of measured data; i is the sequence number of wavelengths; $\lambda_{i}$ represents the measured value of ith wavelength; and $\bar{\lambda }$ is the average value of measured data. Taking the FBG sensor with a central wavelength of 1551.23 nm as an example, the measurement result is shown in Figure 12b.

The system precision is around 0.5 pm by calculation. These results demonstrated that this system has high precision and the static performance is adequate.

### 4.3. The Comparison Analysis of the Interrogation System

TDM was applied to improve the capacity of the interrogation system, with the limitation caused by optical loss of the optical switch. With the consideration of cost and optical loss of the optical switch, 1 × 4 switches were connected for the sake of realizing a multichannel switch of the spectrum. In the experiment, the optical loss was 3.88 dB when four 1 × 4 switches were connected. Considering the insertion loss (1.91 dB), polarization-dependent loss (0.2 dB) of the F–P filter and the additional loss (0.1 dB) of the optical splitter, the theoretical optical power reached by FBG sensors is around 3.9 mW. As the number of cascades was five, incomplete waveforms were detected. Thus, the maximum capacity of this system is 256. In addition to the application of the 800-Hz F–P filter, the frequency can be ensured by application of the FPGA+ARM platform and the maximum wavelength scanning speed measured can reach 800 Hz in the experiment. Thus, the repetition rate of this system is around 3 Hz when the capacity is 256. When the capacity is 8, the repetition rate can reach 100 Hz. The repetition rate is inversely proportional to the capacity. In addition, the static interrogation precision could reach 0.5 pm in the temperature experiment. In this paper, the typical interrogators based on M-Z interferometer [5], WDM+FDM [6] and the latest approaches based on encoding strategy [11], MEMS [24], on-chip AWG [26] are taken into comparison. To assess the performance, the precision, maximum capacity, interrogation and cost of different systems were compared and analyzed. The comparison results are shown in Table 3. Techniques based on M–Z interferometer, MEMS and system proposed in this paper have a precision of around 0.5 pm, which is higher than other systems. Methods based on WDM+FDM, encoding strategy and the approach proposed in this paper can interrogate hundreds of sensors, while other systems’ capacity is less than 10. Furthermore, the proposed system also has a beneficial effect on interrogation range. And it has a low cost required for adopting F–P filter and broadband light source. In conclusion, the proposed system has an optimum comprehensive performance, which can achieve high precision and capacity at the same time.

## 5. Conclusions

In this study, a practical FBG interrogation system based on a FPGA+ARM platform, tunable F–P filter and optical switch was designed and experimentally verified. The comparison of different interrogation theories demonstrated that this system has an optimum comprehensive performance in interrogation precision, capacity and interrogation range. To improve the practical accuracy of the system, we employed a F–P filter to overcome the low precision of a broadband light source. The error of the F–P filter caused by its non-linear characteristic could be compensated with the circuit part. In the soft algorithm, we designed a self-adaption algorithm with peak recognition rate of 100%. The precision of six algorithms under variation of SNR proved the superiority of the algorithm proposed in this paper. For increasing the capacity of interrogation, an optical switch was employed to achieve TDM of the FBG spectrum, which can increase the quantities of FBG sensors interrogated in the system. Through the experiment, at most 256 FBG sensors can be interrogated. To improve the speed of the system, we adopted FPGA to accomplish high-speed control and sampling, while ARM was simultaneously applied to achieve high-speed interrogation calculation. It was demonstrated that a wavelength scanning speed of 800 Hz could be realized. The temperature experiment was conducted to demonstrate the effectiveness of the system. Four FBG sensors were interrogated with a linearity of 0.999 when temperature changed from 0 °C to 100 °C. Static measurement results showed that the precision of the system could reach 0.5 pm.

## Figures and Tables

**Table 1:** The results of Gaussian fit.

Number	Peak1	Peak2	Peak3	Peak4	Peak5	Peak6	Peak7
SSE	0.4499	0.3618	0.3578	0.3676	0.4354	0.435	0.5643
RMSE	0.04512	0.04046	0.04024	0.04078	0.04439	0.04437	0.05053

**Table 2:** Calculation performance of the algorithms.

Algorithms	Execution Time	The Needed Logic Unit
Maximum	1	1
Discrete-time Filter	27.21	12.31
Centroid	0.67	1.72
polynomial fitting	340	1000
Neural Network	25,000	40,000
Self-Adaption	3.15	5.53

**Table 3:** The contrasts of different interrogation systems.

Ref.	Approaches	Precision/pm	Maximum Capacity	Interrogation Range/nm	Cost
[5]	M-Z interferometer	0.47	2	120	middle
[6]	WDM+FDM	3.5	275	70	low
[11]	Encoding strategy	3	3 times than WDM method	100	high
[24]	MEMS	0.5	4	45	low
[26]	On-chip AWG	±10	4	39	high
	This paper	0.5	256	75	low
